# Drought and child vaccination coverage in 22 countries in sub-Saharan Africa: A retrospective analysis of national survey data from 2011 to 2019

**DOI:** 10.1371/journal.pmed.1003678

**Published:** 2021-09-28

**Authors:** Jason M. Nagata, Adrienne Epstein, Kyle T. Ganson, Tarik Benmarhnia, Sheri D. Weiser

**Affiliations:** 1 Department of Pediatrics, University of California, San Francisco, San Francisco, California, United States of America; 2 Department of Epidemiology and Biostatistics, University of California, San Francisco, San Francisco, California, United States of America; 3 Factor-Inwentash Faculty of Social Work, University of Toronto, Toronto, Ontario, Canada; 4 Herbert Wertheim School of Public Health and Human Longevity Science, University of California, San Diego, La Jolla, California, United States of America; 5 Scripps Institution of Oceanography, University of California, San Diego, La Jolla, California, United States of America; 6 Division of HIV, Infectious Diseases and Global Medicine, Department of Medicine, University of California, San Francisco, San Francisco, California, United States of America; Instituto de Salud Global de Barcelona, SPAIN

## Abstract

**Background:**

Extreme weather events, including droughts, are expected to increase in parts of sub-Saharan Africa and are associated with a number of poor health outcomes; however, to the best of our knowledge, the link between drought and childhood vaccination remains unknown. The objective of this study was to evaluate the relationship between drought and vaccination coverage.

**Methods and findings:**

We investigated the association between drought and vaccination coverage using a retrospective analysis of Demographic and Health Surveys data in 22 sub-Saharan African countries among 137,379 children (50.4% male) born from 2011 to 2019. Drought was defined as an established binary variable of annual rainfall less than or equal to the 15th percentile relative to the 29 previous years, using data from Climate Hazards Group InfraRed Precipitation with Station (CHIRPS) data. We evaluated the association between drought at the date of birth and receipt of bacillus Calmette–Guérin (BCG), diphtheria–pertussis–tetanus (DPT), and polio vaccinations, and the association between drought at 12 months of age and receipt of measles vaccination. We specified logistic regression models with survey fixed effects and standard errors clustered at the enumeration area level, adjusting for child-, mother-, and household-level covariates and estimated marginal risk differences (RDs). The prevalence of drought at date of birth in the sample was 11.8%. Vaccination rates for each vaccination ranged from 70.6% (for 3 doses of the polio vaccine) to 86.0% (for BCG vaccination); however, only 57.6% of children 12 months and older received all recommended doses of BCG, DPT, polio, and measles vaccinations. In adjusted models, drought at date of birth was negatively associated with BCG vaccination (marginal RD = −1.5; 95% CI −2.2, −0.9), DPT vaccination (marginal RD = −1.4; 95% CI −2.2, −0.5), and polio vaccination (marginal RD = −1.3; 95% CI −2.3, −0.3). Drought at 12 months was negatively associated with measles vaccination (marginal RD = −1.9; 95% CI −2.8, −0.9). We found a dose–response relationship between drought and DPT and polio vaccinations, with the strongest associations closest to the timing of drought. Limitations include some heterogeneity in findings across countries.

**Conclusions:**

In this study, we observed that drought was associated with lower odds of completion of childhood BCG, DPT, and polio vaccinations. These findings indicate that drought may hinder vaccination coverage, one of the most important interventions to prevent infections among children. This work adds to a growing body of literature suggesting that health programs should consider impacts of severe weather in their programming.

## Abstract 2

Jason M. Nagata and colleagues investigate the association between drought and child vaccination coverage in 22 countries in sub-Saharan Africa, using Demographic and Health Survey data from 2011 to 2019.

## Author summary

**Why was this study done?:**

- Extreme weather events, including droughts, are associated with food insecurity and poor health outcomes, but the link between drought and childhood vaccination has not been studied, to the best of our knowledge.
- Potential mechanisms linking drought to lower childhood vaccination include food insecurity, increased human migration, and erosion of the public health infrastructure.

**What did the researchers do and find?:**

- We combined national survey data from 22 countries in sub-Saharan Africa with publicly available historical rainfall data to estimate drought exposure among 137,379 children born from 2011 to 2019 and estimated the association between drought and childhood vaccination completion.
- Drought was associated with lower odds of completion of childhood bacillus Calmette–Guérin (BCG), diphtheria–pertussis–tetanus (DPT), and polio vaccinations.
- We found a dose–response relationship between drought and DPT and polio vaccinations, with the strongest associations closest to the timing of drought.

**What do these findings mean?:**

- These findings indicate that drought may hinder vaccination coverage, one of the most important interventions to prevent infections among children.
- Public health programs should be prepared to address drought and adverse weather events as a barrier to childhood vaccination efforts in the face of a changing climate.

## Introduction

Childhood vaccination is one of the most successful and cost-effective health interventions known [1]. For instance, polio vaccines have lowered the global incidence of polio by 99% [1–3]. Diphtheria–pertussis–tetanus (DPT) vaccines, as well as measles vaccines, have substantially reduced illness, disability, and death [1,4–6]. Despite these advances, vaccine coverage gaps and disparities remain. For instance, DPT vaccination coverage in low-income countries was 16% lower than in high-income countries in 2010 [1]. There are several explanations for low vaccination coverage, including religious beliefs [7], safety worries [8], barriers due to the healthcare system and providers [9], and war and conflict [10–12]. These barriers may only partially explain the coverage gap. One potential explanation for low vaccination coverage that remains understudied is climate change. Climate change and extreme weather events can negatively impact human health [13,14], including contributing to infectious diseases, malnutrition, chronic diseases, and poor mental health [14,15]. Droughts, defined as a prolonged dry period in the climate cycle, are increasingly common in parts of sub-Saharan Africa, where droughts have recently occurred from 2011 to 2012 (East Africa), from 2014 to 2016 (Southern Africa), and in 2019 (throughout sub-Saharan Africa, exposing 45 million people to drought) [16]. Areas already affected by drought are expected to experience worse droughts [17]. In East Africa, the number of drought events and their duration, frequency, and intensity are expected to increase in Sudan, Tanzania, Somalia, and South Sudan [17].

Drought can lead to reduced agricultural production, which could reduce financial and food security, thus limiting access to healthcare [13,18]. Drought is also associated with poorer child health outcomes, such as acute malnutrition [19] and childhood illness, including fever, diarrhea, and cough [20]. Despite the increase in droughts and the adverse effects associated with them, there is a paucity of studies examining the relationship between droughts and vaccination coverage. Historically, a public health perspective identified child vaccination as one of the highest priority emergency health requirements during the 1973–1974 droughts throughout sub-Saharan Africa [21,22], and case reports have anecdotally reported challenges with vaccination during specific droughts, such as low measles vaccination rates during the 1983–1985 drought in Timbuktu, Mali [23]. A recent qualitative study involving professional stakeholders highlighted the lack of vaccinations for drought-related migrants in Somalia, Kenya, and Ethiopia as a primary healthcare need [24]. However, to the best of our knowledge, there has been no empirical research to date that has rigorously examined the association between drought and vaccination rates. Research on climate change and variability as potential barriers to vaccination coverage in children has important implications for public health programs.

The purpose of this study was to determine the association between drought, using publicly available historical rainfall data from the previous 29 years, and child vaccination, using nationally representative population-based survey data from 22 countries in sub-Saharan Africa from 2011 to 2019. Furthermore, we investigated the magnitude of association between drought at the time of birth and timing of vaccination based on WHO vaccination schedules in the first year of life. We hypothesized that drought would be associated with lower childhood vaccination rates, and that the association between drought and lower vaccination rates would be strongest when the drought and vaccination schedule occurred simultaneously.

## Methods

This study is reported as per the REporting of studies Conducted using Observational Routinely-collected Data (RECORD) guideline (S1 RECORD Checklist).

### Data source and participants

We combined data from Demographic and Health Surveys (DHS) surveys, which are cross-sectional, nationally representative, household-based surveys that use a stratified 2-stage cluster sampling design selecting enumeration areas (EAs) and households within each EA. All women aged 15 to 49 years within the selected households were invited to complete a questionnaire, which included detailed birth histories of all children born in the previous 5 years.

We used surveys that included geolocated information on each EA and that took place during or after 2011, using this year as a cutoff because of the availability of exposure data (see S1 Table for a full list of surveys included in this analysis). We restricted our sample to children born during or after 2011. Full information on outcomes of interest and covariates was also required for inclusion. Mali, Côte d’Ivoire, and Cameroon were excluded from the analysis since drought was a rare occurrence in each of these countries during the study period.

### Measures

The classifications of all variables (drought exposure, vaccination outcomes, and covariates) were defined a priori; however, we did not have a prespecified written protocol for this analysis.

#### Drought

Drought was measured using Climate Hazards Group InfraRed Precipitation with Station (CHIRPS) data, which combine satellite imagery with weather station data to create raster rainfall estimates in millimeters at 0.05 decimal degree resolution from 1981 onward [25]. Annual cumulative precipitation for the 12 months preceding the exposure time point was calculated for each unique exposure time point/EA combination. For bacillus Calmette–Guérin (BCG), DPT, and polio vaccination, drought was assessed at the date of birth. For measles vaccination, drought was assessed at 12 months of age since the WHO vaccination schedule recommends measles vaccination at 9–12 months. We then ranked this quantity of precipitation with those of the prior 29 years and converted this ranking to a percentile; for example, a value of 50% signifies the median level of precipitation in the 30-year period. This use of deviations from long-term precipitation trends is standard in the literature, as it captures weather shocks representing deviations from the norm and, therefore, do not reflect inherent differences in populations that live in dryer or wetter areas [16,26,27]. We generated a binary categorization of drought, defined as annual precipitation in the 12 months prior to the exposure time point that was equal to or lower than 15% of the historical record, reflecting the level of precipitation that impacts GDP and agricultural productivity as defined previously in the literature [26–28]. To determine whether our findings were sensitive to the definition of drought and to identify potential nonlinear relationships between rainfall deviations and vaccination status, we considered a continuous measure of rainfall deviation percentiles using restricted cubic splines, with values closer to 0 representing more severe drought. The number of knots was determined using Akaike’s information criterion.

#### Vaccination outcomes

Vaccination status was assessed during DHS surveys based on either the vaccination card or, when unavailable, mother’s report (21.7% of respondents). We included 4 vaccines that were included in the World Health Organization vaccination schedule [29]: BCG, DPT, polio, and measles (Table 1). For multi-dose vaccinations (DPT and polio), our primary outcome was completion of all 3 doses. For each model, we included only children who should have received the vaccination by the time of the survey. For example, all children were included in models with BCG as an outcome because it is scheduled to be given at birth, but only children ages 6 months and older were included in analyses with DPT as an outcome. In addition, we assessed vaccination status by birth cohort in order to determine if and when associations between drought at birth or 12 months dissipated. The calculation and classifications of drought were specified a priori.

**Table 1:** Description of outcomes included in analyses assessing the association between drought and vaccination.

Vaccine	Recommended timing	Ages included in analyses
Bacillus Calmette–Guérin	At birth	All ages
Diphtheria–pertussis–tetanus		
Dose 1	6 weeks	2 months and older
Dose 2	10 weeks	4 months and older
Dose 3	14 weeks	6 months and older
All 3 doses		6 months and older
Polio		
Dose 1	6 weeks	2 months and older
Dose 2	10 weeks	4 months and older
Dose 3	14 weeks	6 months and older
All 3 doses		6 months and older
Measles	9 or 12 months	12 months and older

#### Covariates

We included several sociodemographic variables a priori that have theoretical and empirical associations with vaccination status without being induced by droughts [30–32]. These include child-level variables (sex, birth month, and birth order), mother-level variables (age at time of survey [15–19, 20–29, 30–39, or 40–49 years], a binary indicator of respondent literacy [literate versus not literate, measured by whether the respondent could read a sentence presented to them during the survey], education [none, primary, secondary, or higher), and marital status [never married, currently married/living with partner, divorced/separated, or widowed]), and household-level variables (wealth index as defined by DHS using a principal components analysis [33], urban residence [binary indicator according to the definition of urban and rural in the survey country], and household size [1–3, 4–5, 6–7, or 8+ individuals] [34]).

### Statistical analysis

To assess the association between drought and vaccination status, a series of multivariable logistic regression models were specified for each outcome. We then derived marginal risk differences (RDs) using Stata’s *margins* command, which estimates effects by first deriving predictions for each observation in the sample as if it was present in each level of the exposure (no drought and drought). For each level of exposure, each individual’s marginal effect is then estimated by subtracting the predicted probability of the outcome under the reference group (no drought) from the predicted probability under drought. The effect estimate is then averaged across all observations. In all models, we included survey fixed effects to control for time-fixed country-level differences that are measured or not, such as cultural norms and health behaviors, sociodemographic characteristics, and economies, and we included robust standard errors clustered at the EA level. We first ran the models with only survey-level fixed effects and the drought indicator variable, and subsequently added in the covariates. All analyses were carried out in Stata version 14 and R version 3.4.

### Ethical approval

DHS obtains informed and voluntary consent from survey participants, and permission to use DHS data was obtained from the DHS program. Specific approval for this de-identified secondary data analysis was not required.

## Results

In sum, 137,379 children were included in the analysis (Table 2; see S1 Fig for how the sample was determined). Approximately half (50.4%) of the children were male, and most (87.6%) were first-born. Over half (52.0%) of the mothers were age 20–29 years at the time of survey, over two-thirds (83.8%) were married, less than half (44.8%) were literate, and 69.6% had at least some formal schooling. All household wealth categories were represented, with the smallest proportion being in the richest category (15.1%). Most (70.0%) households were in rural areas. The most prevalent household size was 4–5 individuals (31.7%). The proportion of children vaccinated was highest for BCG (86.1%) and lowest for polio (70.7%). The distributions of vaccinations by vaccine and country are presented in S2 Table.

**Table 2:** Descriptive statistics of children born between 2011 and 2019 included in the analysis (*n* = 137,379).

Variable	Percent (*n*)
**Infant-level variables**	
Male	50.4 (69,264)
Birth order	
1	87.6 (120,312)
2	11.9 (16,396)
3	0.5 (647)
4	0.02 (21)
5	0.00 (3)
**Mother-level variables**	
Age (years)	
15–19	8.9 (12,286)
20–24	25.4 (34,889)
25–29	26.6 (36,469)
30–34	19.6 (26,855)
35–39	12.8 (17,581)
40–44	5.4 (77,473)
45–49	1.3 (1,826)
Marital status	
Never married	8.9 (12,254)
Currently married/living with partner	83.8 (115,102)
Divorced/separated	5.9 (8,159)
Widowed	1.4 (1,864)
Literate	44.8 (61,514)
Education	
None	30.4 (41,789)
Primary	42.0 (57,706)
Secondary	24.7 (33,919)
Higher	2.9 (3,965)
**Household-level variables**	
Household wealth	
Poorest	25.6 (35,100)
Poorer	21.8 (29,923)
Middle	19.9 (27,286)
Richer	17.8 (24,386)
Richest	15.1 (20,684)
Urban (versus rural)	30.0 (41,594)
Household size	
1–3	12.6 (17,274)
4–5	31.7 (43,485)
6–7	26.8 (36,829)
8+	29.0 (39,791)
**Vaccination outcomes**	
BCG	86.1 (118,290)
DPT (3 doses)	73.5 (83,734)
Polio (3 doses)	70.7 (80,544)
Measles	77.4 (69,768)

BCG, bacillus Calmette–Guérin; DPT, diphtheria–pertussis–tetanus.

**Fig 1:**
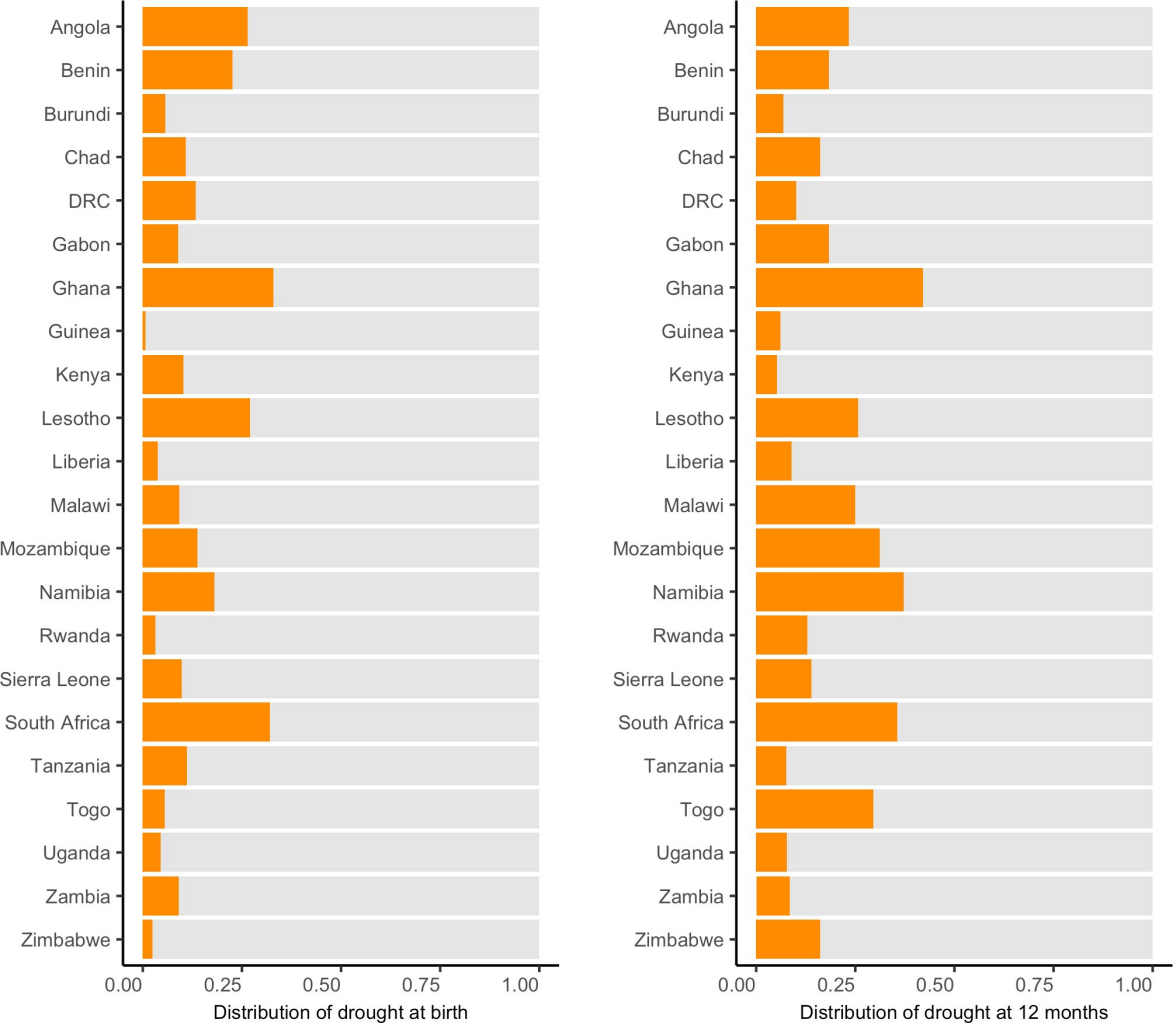
Distribution of drought at birth and 12 months age. The proportions of observations in drought are represented by orange bars. DRC, Democratic Republic of the Congo.

Fig 1 shows the percentage of observations in drought around birth and at age 12 months by country. Drought at birth was most common in South Africa (32.1% of births) and least common in Guinea (0.7% of births).

Table 3 shows the odds ratios for the relationship between drought and receipt of vaccination for BCG, DPT (3 doses), polio (3 doses), and measles. In models adjusting for child sex, birth month, and birth order; mother’s age, literacy, education, and marital status; and household wealth index, household size, and urban residence, drought at date of birth was associated with a 1.5 percentage point risk reduction for BCG vaccination (marginal RD = −1.5; 95% CI −2.2, −0.9), a 1.4 percentage point risk reduction for DPT vaccination (marginal RD = −1.4; 95% CI −2.3, −0.5), and a 1.3 percentage point risk reduction for polio vaccination (marginal RD = −1.3; 95% CI −2.3, −0.3), compared to children born at dates when drought was not present. Drought at 12 months was associated with a 1.9 percentage point risk reduction for measles vaccination (marginal RD = −1.9; 95% CI −2.8, −0.9) compared to non-drought. In sensitivity analyses, we removed the literacy covariate to assess the potential for multicollinearity with education, but findings were qualitatively similar (S3 Table). Models that included rainfall deviation percentiles modeled nonlinearly as cubic splines revealed nonlinear relationships between deviations and vaccination (S2 Fig). For each vaccine, the probability of vaccination remained flat below approximately the 35th percentile of historical precipitation, above which the probability of vaccination increased. The nonlinear associations shown in S2 Fig demonstrate that associations between more severe drought (rainfall percentile deviation values closer to 0) and vaccination were similar to associations between moderate drought (rainfall percentile deviation values closer to the 35th percentile) and vaccination.

**Table 3:** Associations between drought and vaccination among children born in 2011–2019 (*n* = 137,379).

Exposure	Outcome—marginal risk difference (95% CI)							
	BCG (among all children), *n* = 137,379		DPT (3 doses, among children 6 months and up), *n* = 113,987		Polio (3 doses, among children 6 months and up), *n* = 113,987		Measles (among children 12 months and up), *n* = 90,201	
	Unadjusted	Adjusted	Unadjusted	Adjusted	Unadjusted	Adjusted	Unadjusted	Adjusted
Drought^†^	−1.9 (−2.6, −1.2)	−1.5 (−2.2, −0.9)	−1.6 (−2.6, −0.7)	−1.4 (−2.2, −0.5)	−1.6 (−2.6, −0.6)	−1.3 (−2.3, −0.3)	−2.1 (−3.1, −1.1)	−1.9 (−2.8, −0.9)

Coefficients are presented as marginal risk differences in percentage points derived from logistic regression models, with 95% confidence intervals in parentheses. The unadjusted model includes country-level fixed effects. The adjusted model includes child sex, birth month, and birth order; mother’s age (15–19, 20–29, 30–39, or 40–49 years), mother’s literacy (literate versus not literate), mother’s education, and mother’s marital status; and household wealth index (quintiles), household size, and urban residence. Standard errors are clustered at the enumeration area level.

^†^For BCG, DPT, and polio vaccination, the exposure period for drought was the 12 months prior to the date of birth. For measles, the exposure period was the 12 months prior to the child’s first birthday.

BCG, Bacillus Calmette–Guérin; DPT, diphtheria–pertussis–tetanus.

**Fig 2:**
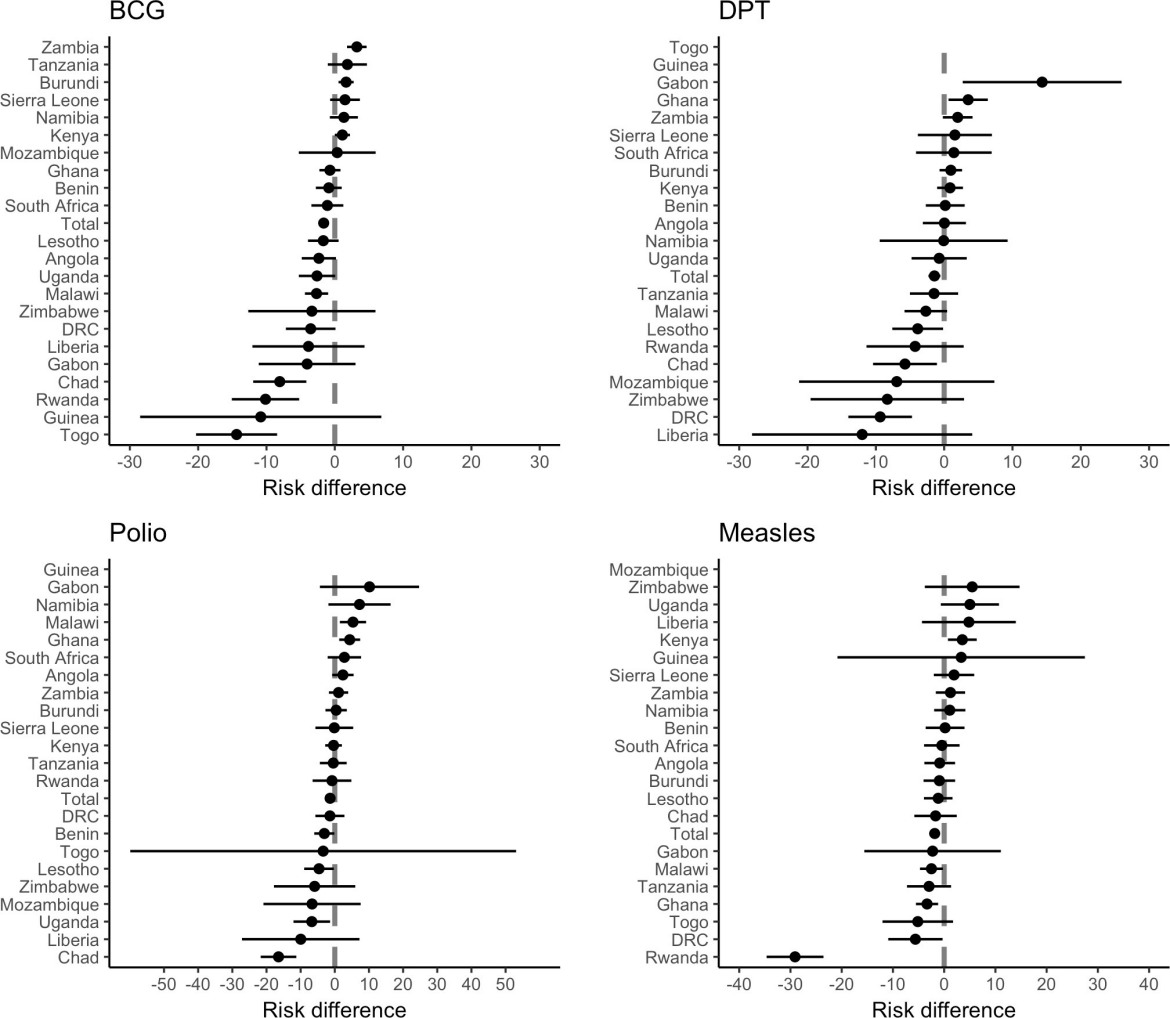
Country-specific adjusted associations between drought and vaccination. (A) BCG vaccination; (B) 3 doses of DPT vaccination; (C) 3 doses of polio vaccination; (D) measles vaccination. Models are adjusted for child sex, birth month, and birth order; mother’s age (15–19, 20–29, 30–39, or 40–49 years), mother’s literacy (literate versus not literate), mother’s education, and mother’s marital status; and household wealth index (quintiles), household size, and urban residence. Standard errors are clustered at the enumeration area level. Associations are presented as marginal risk differences in percentage points and 95% confidence intervals. Results are not shown for countries with insufficient variation in exposure/outcome data. BCG, Bacillus Calmette–Guérin; DPT, diphtheria–pertussis–tetanus; DRC, Democratic Republic of the Congo.

Fig 2 shows the associations between drought and full vaccination for BCG, DPT, polio, and measles by country. Seven countries (Togo, Rwanda, Chad, Democratic Republic of the Congo, Malawi, Uganda, and Angola) demonstrated negative associations between drought at birth and BCG vaccination, three countries (Democratic Republic of the Congo, Chad, and Lesotho) demonstrated negative associations between drought at birth and 3 doses of DPT vaccination, 4 countries (Chad, Uganda, Lesotho, and Benin) demonstrated negative associations between drought at birth and 3 doses of polio vaccination, and 4 countries (Rwanda, Democratic Republic of the Congo, Ghana, and Malawi) demonstrated negative associations between drought at 12 months and measles vaccination. These country-level analyses also yielded instances when drought was positively associated with vaccination. In Zambia and Kenya, drought was positively associated with BCG vaccination. In Gabon and Burundi, drought was positively associated with DPT vaccination. In Malawi and Ghana, drought was positively associated with polio vaccination. Finally, in Kenya, drought was positively associated with measles vaccination. Due to this heterogeneity, we also specified models using mixed effects regression (with random intercepts at the survey and EA levels). Findings were qualitatively consistent (in magnitude and direction) across specifications (S4 Table).

Table 4 shows the association between drought at date of birth and vaccination for each dose of DPT and polio vaccination. These results show similar patterns; the negative association between drought at birth and vaccination status is strongest for the first dose and is attenuated at subsequent doses. These findings were echoed in analyses assessing the relationship between drought at birth or 12 months and vaccination by birth cohort (S3 Fig). Drought at birth was negatively associated with receipt of DPT and polio vaccination among children under 24 months, after which the associations attenuated. Drought at 12 months was negatively associated with measles vaccination among children aged 12 months to less than 24 months, but not among birth cohorts 24 months or older. Finally, drought at birth was negatively associated with BCG vaccination among children aged 12 months to less than 24 months, but not among children aged less than 12 months and those aged 24 months or older.

**Table 4:** Adjusted associations between drought at date of birth and each dose of DPT and polio vaccination.

Dose	Marginal risk difference (95% CI)			
	DPT		Polio	
	Unadjusted	Adjusted	Unadjusted	Adjusted
Dose 1	−2.6 (−3.3, −1.9)	−2.3 (−3.0, −1.7)	−1.9 (−2.5, −1.2)	−1.6 (−2.2, −1.0)
Dose 2	−2.1 (−2.9, −1.3)	−1.9 (−2.6, −1.1)	−1.6 (−2.4, −0.9)	−1.4 (−2.1, −0.7)
Dose 3	−1.5 (−2.5, −0.7)	−1.3 (−2.2, −0.4)	−1.7 (−2.6, −0.7)	−1.4 (−2.3, −0.4)

Coefficients are presented as marginal risk differences in percentage points derived from logistic regression models, with 95% confidence intervals in parentheses. The unadjusted model includes country-level fixed effects. The adjusted model includes child sex, birth month, and birth order; mother’s age (15–19, 20–29, 30–39, or 40–49 years), mother’s literacy (literate versus not literate), mother’s education, and mother’s marital status; and household wealth index (quintiles), household size, and urban residence. Standard errors are clustered at the enumeration area level.

DPT, diphtheria–pertussis–tetanus.

## Discussion

In this nationally representative cross-sectional study of 22 countries in sub-Saharan Africa, drought was associated with lower odds of completion of childhood BCG, DPT, and polio vaccination. The association between drought at the date of birth and DPT and polio vaccination was strongest for the first dose (closest to timing of drought) and attenuated, but was still substantial for subsequent doses. This study contributes to the limited but growing literature documenting an association between drought and poor health outcomes. These findings have important implications as childhood vaccination is one of the most widespread and successful public health interventions available to prevent infections.

To the best of our knowledge, this is the first study to systematically assess the association between drought and vaccination. Prior case studies have reported low vaccination rates during historical drought periods. For instance, a 47% measles vaccination coverage rate was reported during the 1983–1985 drought in Timbuktu, Mali [23]. Measles vaccination was identified as a top public health priority during droughts in 1973–1974 throughout sub-Saharan Africa and in 2011 in Somalia [21,22]. It is also noteworthy that we found a dose–response relationship between drought and vaccination, with the strongest association closest to the timing of drought. One exception was the lack of association between drought and BCG vaccination among children less than 12 months; this may be due to the fact that these children received their BCG vaccination right at the time of birth (as per WHO recommendations). If drought impacts vaccination through barriers to clinic access, it may play a smaller role in hindering vaccination among infants already at the hospital immediately after birth. Prior studies have found an association between drought and childhood illness, including fever, diarrhea, and cough, in sub-Saharan Africa [20]. Parents may avoid vaccination when their children are ill due to perceptions that the vaccination could exacerbate illness, exacerbate symptoms, or produce pain [35]. Alternatively, lower vaccination rates lead to higher susceptibility to infectious diseases such as tuberculosis, diphtheria, pertussis, tetanus, and polio, which in turn could lead to illness symptoms such as fever, cough, and diarrhea [1].

The association between drought and lower vaccination rates may be explained by several mechanisms, including increased food insecurity, intimate partner violence, poorer mental health, increased human migration, avoidance of vaccination due to illness, and erosion of the public health infrastructure [13]. First, drought can detrimentally affect agricultural and crop production, thus leading to food insecurity and financial instability [14,18]. With fewer financial and food resources, parents may be less able to afford healthcare [36–38] or pay for transportation to travel to clinics for vaccinations [39]. Second, drought and subsequent financial and food insecurity can worsen mental health through emotional stress, depression, anxiety, and increased alcohol consumption [40–42]. Drought is also associated with increases in intimate partner violence, which may impact parental mental health [16]. Parents with poorer mental health may be less likely to take their children to healthcare visits. Third, drought may lead to human migration as a last resort for households [13,43]. In the context of migration, families may be less able to access healthcare in new geographical settings, a possibility that was highlighted in a recently published study [24]. Finally, extreme weather events, including droughts, can detrimentally impact the healthcare system and infrastructure. For instance, drought and heat waves can increase risk for wildfires [13,44]. Drought can also affect the production of electricity and disrupt power supplies by interfering with heating and cooling systems at power plants, which could affect the ability to store vaccines at recommended temperatures using refrigeration [13]. Future research could investigate potential mechanisms by which drought may negatively impact vaccination rates.

Limitations of this study should be noted. There was heterogeneity in findings across countries. However, some of the countries where drought was associated with higher vaccination may be in regions that typically experience heavy rains, so drought (based on our definition) in this context may have been less disruptive than heavy rains. There may be inconsistencies in data collection across countries and across years even though DHS does make attempts to achieve standardization in data collection procedures across locations and time points. The distribution of satellite data and ground stations used by the CHIRPS precipitation dataset may not be consistent, and some countries may have less accurate precipitation data than others, thus leading to the potential for misclassification of drought in some settings. However, by classifying drought as a percentile of annual precipitation over 30 years, rather than using absolute rainfall estimates, we reduced the potential for misclassification. In addition, CHIRPS incorporates a number of data checks and quality control measures to ensure that station anomalies and inconsistencies are corrected [25]. There is potential for reporting and recall bias given the self-reported nature of some of the DHS variables; however, a majority (78.3%) of the vaccination data were derived from vaccination cards.

Despite these limitations, important strengths of the study include the inclusion of 22 countries with nationally representative data of over 137,000 children, representing varying agricultural systems, environments, and sociodemographic characteristics. Furthermore, we aggregated data collection over 9 years, which represented a wide range of drought conditions. There was low potential for confounding as drought was defined as precipitation relative to the previous 29 years. Therefore, the drought exposure should be independent from potential confounding variables. Finally, vaccination was primarily defined based on vaccine records rather than self-report.

This study contributes to the growing literature on climate change and health, identifying an association between drought and lower child vaccination rates. Vaccinations are one of the most impactful interventions to prevent childhood illness; thus, identifying drought as a barrier to vaccination has important public health and clinical implications. Given the anticipated acceleration of droughts due to climate change, understanding the barriers to vaccination during drought periods and developing interventions to address these barriers are an important area of future research. National and regional medical and public health systems will need funding and planning to address the healthcare needs of communities impacted by climate change. Global, national, and local stakeholders should be prepared to focus their attention to increase vaccination efforts in the face of the adverse weather events of a changing climate.

## Supporting information

S1 RECORD Checklist RECORD checklist. (DOCX)

S1 Fig Flowchart depicting how the final analytic sample was selected. (PDF)

S2 Fig Nonlinear relationships between rainfall deviations and vaccination outcomes. (PDF)

S3 Fig Associations between drought at birth (for BCG, DPT, and polio vaccination) and 12 months (for measles vaccination) and vaccination status by birth cohort. (PDF)

S1 Table Sample size of each survey included in the analysis. (PDF)

S2 Table Vaccine coverage in the sample by survey. (PDF)

S3 Table Associations between drought and vaccination among children born in 2011–2019 (*n* = 137,379) with the covariate mother’s literacy removed to assess for multicollinearity with mother’s education. (PDF)

S4 Table Associations between drought and vaccination among children born in 2011–2019 (*n* = 137,379) with random intercepts at the survey and enumeration area level. (PDF)

## Abbreviations

CHIRPS: Climate Hazards Group InfraRed Precipitation with Station
BCG: bacillus Calmette–Guérin
DHS: Demographic and Health Surveys
DPT: diphtheria–pertussis–tetanus
EA: enumeration area
RD: risk difference
