# Supplementary figures and images for: Drought and child vaccination coverage in 22 countries in sub-Saharan Africa: A retrospective analysis of national survey data from 2011 to 2019
Source: PLoS Med. 2021 Sep 28;18(9):e1003678. doi: 10.1371/journal.pmed.1003678 (PMC8478213; doi:10.1371/journal.pmed.1003678)

**Figure S1. Flow chart depicting how the final analytic sample was selected**

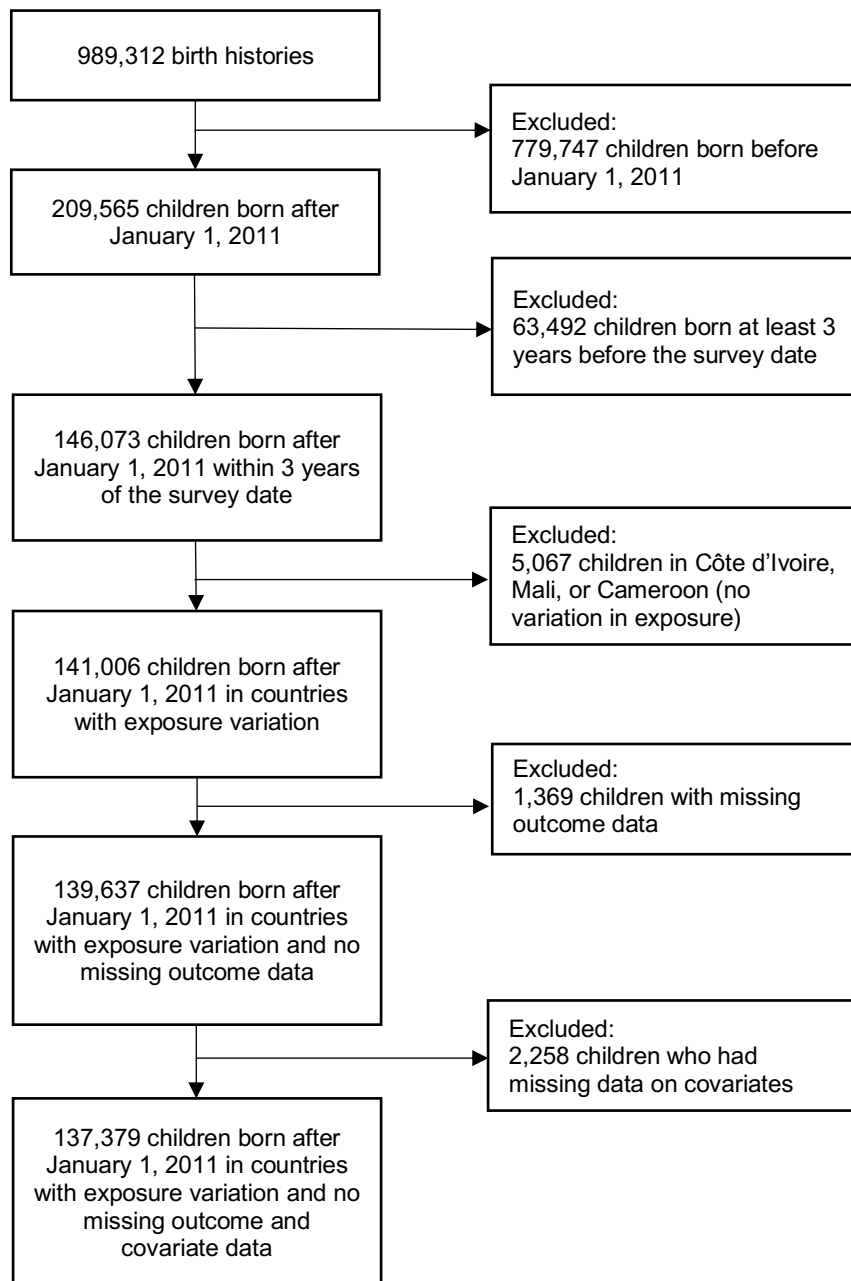

Supplement: S1 Fig — (PDF) [file pmed.1003678.s002.pdf]
