# Supplementary material for: Drought and child vaccination coverage in 22 countries in sub-Saharan Africa: A retrospective analysis of national survey data from 2011 to 2019
Source: PLoS Med. 2021 Sep 28;18(9):e1003678. doi: 10.1371/journal.pmed.1003678 (PMC8478213; doi:10.1371/journal.pmed.1003678)
Supplement: S2 Fig — (PDF) [file pmed.1003678.s003.pdf]

Figure S2. Non-linear relationships between rainfall deviations and vaccination outcomes. Rainfall deviation percentiles were modeled using restricted cubic splines.

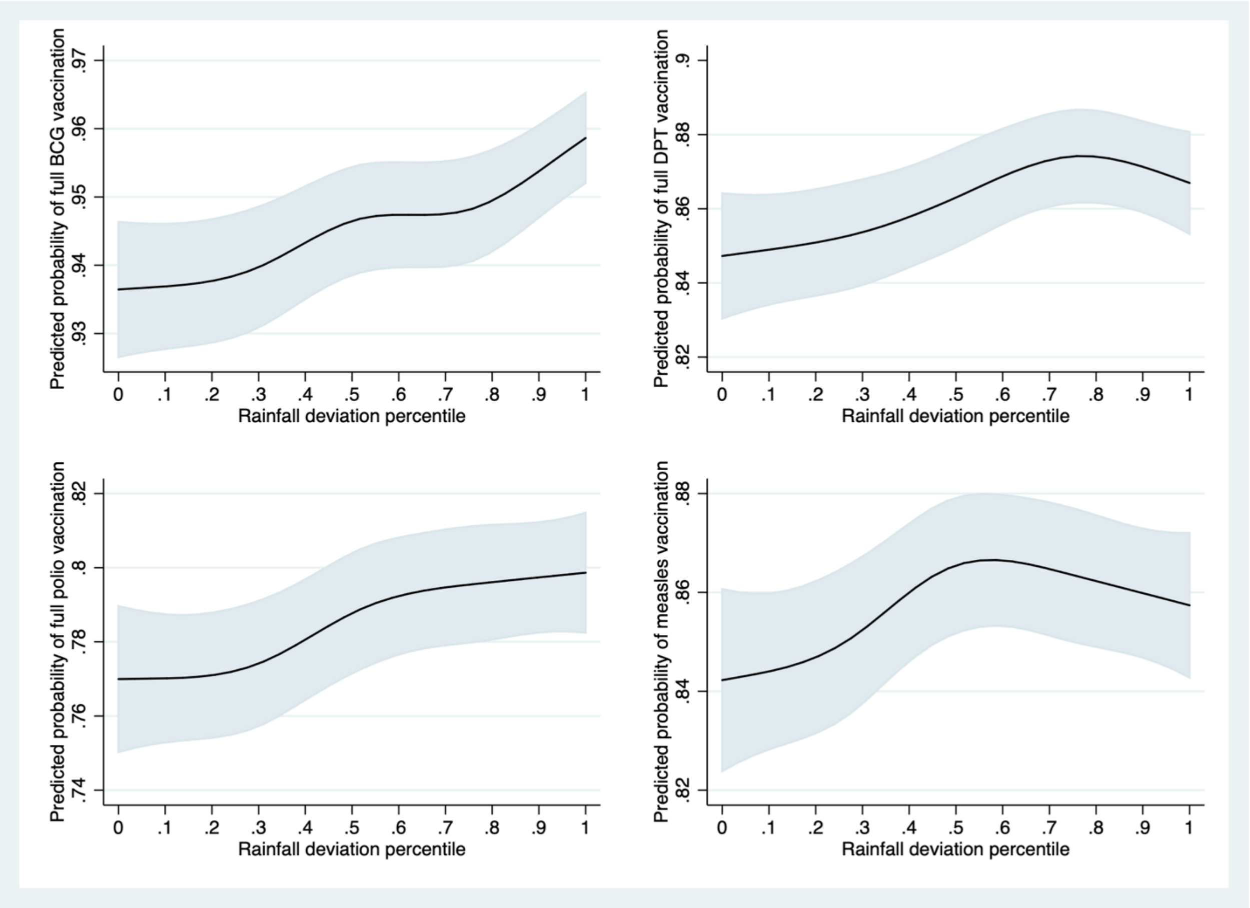

BCG: Bacillus Calmette-Guérin; DPT: Diphtheria-pertussis-tetanus
