# Supplementary material for: Drought and child vaccination coverage in 22 countries in sub-Saharan Africa: A retrospective analysis of national survey data from 2011 to 2019
Source: PLoS Med. 2021 Sep 28;18(9):e1003678. doi: 10.1371/journal.pmed.1003678 (PMC8478213; doi:10.1371/journal.pmed.1003678)
Supplement: S3 Fig — (PDF) [file pmed.1003678.s004.pdf]

Figure S3. Associations between drought at birth (for BCG, DPT, and polio vaccination) and 12 months (for measles vaccination) and vaccination status by birth cohort

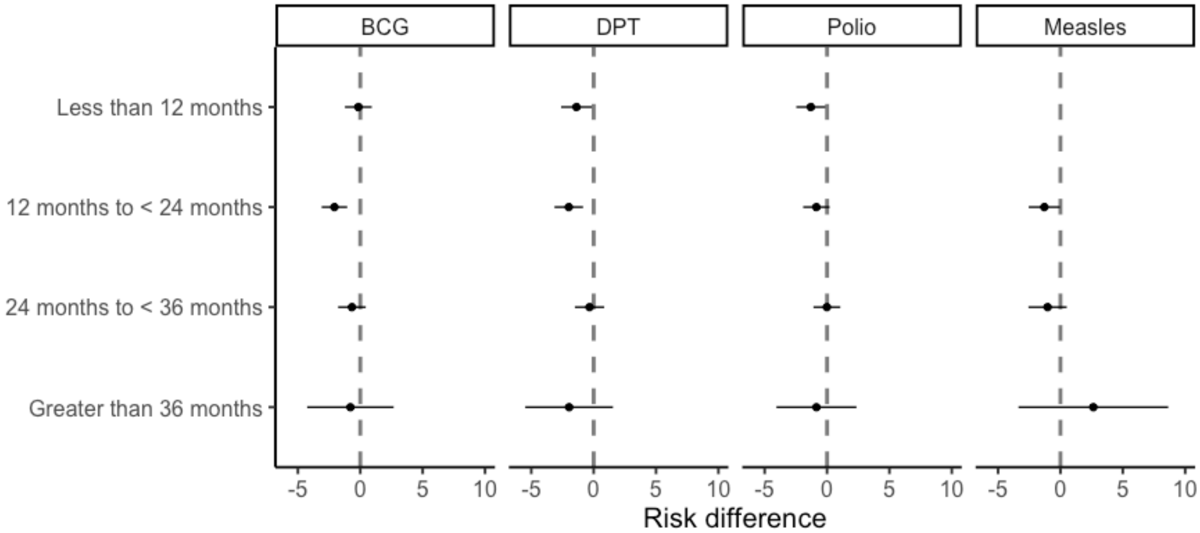

BCG: Bacillus Calmette-Guérin; DPT: Diphtheria-pertussis-tetanus
