# Supplementary material for: Drought and child vaccination coverage in 22 countries in sub-Saharan Africa: A retrospective analysis of national survey data from 2011 to 2019
Source: PLoS Med. 2021 Sep 28;18(9):e1003678. doi: 10.1371/journal.pmed.1003678 (PMC8478213; doi:10.1371/journal.pmed.1003678)
Supplement: S1 Table — (PDF) [file pmed.1003678.s005.pdf]

Table S1. Sample size of each survey included in the analysis	
**Survey**	**N**
Angola 2015-16	8,401
Benin 2017-18	7,395
Burundi 2016-17	7,584
Chad 2014-15	12,603
Democratic Republic of Congo 2013-14	9,478
Gabon 2012	1,597
Ghana 2014	4,338
Guinea 2012	2,409
Kenya 2014	14,448
Lesotho 2014	2,315
Liberia 2013	3,431
Mozambique 2011	1,462
Malawi 2015-16	9,827
Namibia 2013	2,566
Rwanda 2014-15	6,341
Sierra Leone 2013	5,751
South Africa 2016	1,941
Tanzania 2015-16	6,087
Togo 2013-14	4,089
Uganda 2016	8,715
Zambia 2013-14	7,455
Zambia 2018	5,663
Zimbabwe 2015	3,483
**Total**	**137,379**
