# Supplementary material for: Drought and child vaccination coverage in 22 countries in sub-Saharan Africa: A retrospective analysis of national survey data from 2011 to 2019
Source: PLoS Med. 2021 Sep 28;18(9):e1003678. doi: 10.1371/journal.pmed.1003678 (PMC8478213; doi:10.1371/journal.pmed.1003678)
Supplement: S2 Table — (PDF) [file pmed.1003678.s006.pdf]

Table S2. Vaccine coverage in the sample by survey

	BCG		DPT		Polio		Measles	
**Survey**	**N**	**%**	**N**	**%**	**N**	**%**	**N**	**%**
Angola 2015-16	5,592	66.6	2,354	34.2	2,451	35.6	2,864	52.4
Benin 2017-18	6,369	86.1	4,451	71.5	4,025	64.7	3,216	67.7
Burundi 2016-17	7,352	96.9	6,183	96.3	5,817	90.6	4,782	94.4
Chad 2014-15	6,442	51.1	3,040	28.2	5,059	46.9	5,184	56.8
Democratic Republic of Congo 2013-14	6,940	73.2	4,182	54.4	4,703	61.2	4,318	71.5
Gabon 2012	1,340	83.9	153	15.9	390	40.6	202	69.2
Ghana 2014	4,133	95.3	3,194	85.6	3,048	81.7	2,857	90.8
Guinea 2012	1,827	75.8	735	43.8	765	45.5	573	58.6
Kenya 2014	13,611	94.2	11,075	87.7	10,082	79.8	9,129	86.5
Lesotho 2014	2,178	94.1	1,690	85.6	1,453	73.6	1,508	92.7
Liberia 2013	2,990	87.1	1,760	65	1,782	65.9	1,336	72.2
Mozambique 2011	1,206	82.5	225	56.4	224	56.1	N/A	N/A
Malawi 2015-16	9,480	96.5	7,643	92.5	6,655	80.5	6,145	92.8
Namibia 2013	2,461	95.9	1,720	84.9	1,549	76.5	1,383	91.2
Rwanda 2014-15	6,239	98.4	5,533	98.3	5,410	96.1	1,905	40.4
Sierra Leone 2013	5,388	93.7	3,538	77	3,532	76.9	2,868	83.3
South Africa 2016	1,819	93.7	1,118	70	1,225	76.7	1,155	90.5
Tanzania 2015-16	5,637	92.6	4,482	87.7	4,160	81.4	3,565	86.9
Togo 2013-14	3,811	93.2	2,876	82.7	2,512	72.2	2,176	79.3
Uganda 2016	8,259	94.8	5,696	78	4,708	64.5	4,788	83.2
Zambia 2013-14	6,746	90.5	5,338	85.2	4,793	76.5	4,357	87.5
Zambia 2018	5,331	94.1	4,299	90.8	3,766	79.5	3,479	91.6
Zimbabwe 2015	3,139	90.1	2,449	84.1	2,435	83.6	1,978	84.4

BCG: Bacillus Calmette-Guérin; DPT: Diphtheria-pertussis-tetanus
