# Supplementary material for: Drought and child vaccination coverage in 22 countries in sub-Saharan Africa: A retrospective analysis of national survey data from 2011 to 2019
Source: PLoS Med. 2021 Sep 28;18(9):e1003678. doi: 10.1371/journal.pmed.1003678 (PMC8478213; doi:10.1371/journal.pmed.1003678)
Supplement: S3 Table — (PDF) [file pmed.1003678.s007.pdf]

Table S3. Associations between drought and vaccination among children born from 2011-2019 (n = 137,379) with mother's literacy covariate removed to assess for multicollinearity with education

	Outcome							
	BCG (among all children) n = 137,379		DPT (3 doses, among children 6 months and up) n = 113,987		Polio (3 doses, among children 6 months and up) n = 113,987		Measles (among children 12 months and up) n = 90,201	
Exposure	Unadjusted	Adjusted	Unadjusted	Adjusted	Unadjusted	Adjusted	Unadjusted	Adjusted
Drought†	-1.9 (-2.6, -1.2)	-1.5 (-2.2, -0.9)	-1.6 (-2.6, -0.7)	-1.4 (-2.3, -0.5)	-1.6 (-2.6, -0.6)	-1.3 (-2.3, -0.3)	-2.1 (-3.1, -1.1)	-1.9 (-2.8, -0.9)

†For BCG, DPT, and polio vaccination, the exposure period for drought was the 12 months prior to the date of birth. For measles, the exposure period was the 12 months prior to the child's first birthday.
Coefficients are presented as marginal risk differences in percentage points derived from mixed effect linear probability models with 95% confidence intervals in parentheses. The unadjusted model includes survey-level fixed effects. The adjusted model includes child sex, birth month, and birth order; mother's age (15-19, 20-29, 30-39, 40-49), mother's education, and mother's marital status; and household wealth index (quintiles), household size, and urban residence.
BCG: Bacillus Calmette-Guérin; DPT: Diphtheria-pertussis-tetanus
